# Contact Toxicity and Repellency of the Main Components From the Essential Oil of *Clausena anisum-olens* Against Two Stored Product Insects

**DOI:** 10.1093/jisesa/iev071

**Published:** 2015-07-01

**Authors:** Chun Xue You, Hai Yan Jiang, Wen Juan Zhang, Shan Shan Guo, Kai Yang, Ning Lei, Ping Ma, Zhu Feng Geng, Shu Shan Du

**Affiliations:** ^1^Beijing Key Laboratory of Traditional Chinese Medicine Protection and Utilization, Beijing Normal University, Haidian District, Beijing 100875, China; ^2^College of Pharmacy, Liaoning University of Traditional Chinese Medicine, Dalian, 116600, China; ^3^Department of Pharmacy General Hospital of Second Artillery, PLA, Haidian District, Beijing 100088, China; ^4^Analytical and Testing Center, Beijing Normal University, Beijing 100875, China

**Keywords:** *Clausena anisum-olens*, contact toxicity, repellency, *Lasioderma serricorne*, *Liposcelis bostrychophila*

## Abstract

The essential oil of *Clausena anisum-olens* (Blanco) Merr. showed strong contact toxicity and repellency against *Lasioderma serricorne* and *Liposcelis bostrychophila* adults. The components of the essential oil obtained by hydrodistillation were determined by gas chromatography-mass spectrometry. It was found that the main components were myristicin (36.87%), terpinolene (13.26%), p-cymene-8-ol (12.38%), and 3-carene (3.88%). Myristicin and p-cymene-8-ol were separated by silica gel column chromatography, and their molecular structures were confirmed by means of physicochemical and spectrometric analysis. Myristicin and p-cymene-8-ol showed strong contact toxicity against *L. serricorne* (LD_50_ = 18.96 and 39.68 μg per adult) and *Li. bostrychophila* (LD_50_ = 20.41 and 35.66 μg per adult). The essential oil acting against the two grain storage insects showed LD_50_ values of 12.44 and 74.46 μg per adult, respectively. Myristicin and p-cymene-8-ol have strong repellent toxicity to *Li. bostrychophila*.

The cigarette beetle, *Lasioderma serricorne* (F.) (Coleoptera: Anobiidae), is one of the major pests of durable grain commodities, spices, and stored tobaccos. Its larvae cause feeding damage mostly to commodities and its adults rarely feed and are good fliers (Mahroof 2013). The booklouse, *Liposcelis bostrychophila* Badonnel (Psocoptera: Liposcelididae), has a worldwide distribution. It is popularly found in stored-product grains and amylaceous products (Nayak et al. 2005). They have been regarded as the secondary pests, due to their small size and the great damages in cereal grains (Turner 1998). Currently, psocid is perhaps the most important category of emerging pests in stored grains and related commodities in China (Li et al. 2011b).

Control of stored product insects relies heavily on the use of synthetic insecticides and fumigants, which has led to problems such as disturbances of the environment, increasing costs of application, pest resurgence, pest resistance to pesticides, and lethal effects on nontarget organisms in addition to direct toxicity to users (Isman 2004). The widespread extensive use of synthetic insecticides has led to many negative consequences, resulting in increasing attention being given to natural products (Zhang et al. 2011). Botanical pesticides have the advantage of providing novel modes of action against insects that can reduce the risk of cross-resistance and offer new leads for design of target-specific molecules (Li et al. 2011a). Investigations in several countries confirmed that some plant essential oils not only repelled insects but also possessed contact and fumigant toxicities against stored product pests. Moreover, these plant essential oils can exhibit feeding inhibition or harmful effects on the reproductive system of insects (Isman 2006). Essential oils and their constituents have been evaluated for repellent and insecticidal activities against stored product insects or mites, and some of them are quite promising in the development of natural repellents or insecticides (Lee et al. 2006, Liu et al. 2007, Zoubiri and Baaliouamer 2011, Liang et al. 2013). During the screening program for new agrochemicals from Chinese medicinal herbs, essential oil derived from dried rhizome of *Clausena anisum-olens* (Blanco) Merr. (Family: Rutaceae) was found to possess strong insecticidal and repellent activities against the stored product insects.

*C. anisum-olens* is a shrub that grows wildly and is cultivated in Southeast Asia countries such as Philippines and South China. Its aerial parts have been used for the treatment of dysentery and arthritis (Institutum Botanicum Kunmingenge Academiae Sinicae 2001). To date, coumarins and cyclopeptides were isolated from the leaves and twigs of this plant (Wang et al. 2008a,b, 2009a,b). The compositions of *C. anisum-olens* essential oil were also analyzed previously (Molino 2000, Su and Liang 2011). However, there is no report on isolation of *C. anisum-olens* essential oil and insecticidal activity of the essential oil against stored product insects.

## Materials and Methods

### Chinese Medicinal Herb and Extractions

Dried leaves of *C. anisum-olens* (3.0 kg) were collected in May 2013 from Yulin City, Guangxi Autonomous Region, (Guangxi 537000, China, 22.38° N latitude and 106.42° E longitude). A voucher specimen (BNU-HSL-Dushushan- 2013-05-29-017) was deposited in the College of Resources Sciences, Beijing Normal University, Beijing 100875, China. The dried leaves were ground to a powder. The hydrodistillation of the sample was performed in a modified Clevenger-type apparatus for 6 h. The raw oil was extracted with hexane and then dried with anhydrous Na_2_SO_4_. The essential oil was stored in airtight containers in a refrigerator at 4°C.

### Insects

The insects were obtained from laboratory cultures maintained for the last 2 yr in the dark, in incubators at 29–30°C, and 70–80% relative humidity. *L. serricorne* was reared on wheat flour mixed with yeast (10:1, w/w) at 12–13% moisture content, and *Li. bostrychophila* was reared on a 1:1:1 mixture, by mass, of milk powder, active yeast, and flour. Adult insects used in the experiments were 1–2 wk old.

### Gas Chromatography and Mass Spectrometry

Components of the essential oil were separated and identified by gas chromatography-mass spectrometry (GC-MS) on an Agilent 6890N apparatus equipped with FID and an Agilent 5973N mass selective detector. They were equipped with a flame ionization detector (280°C) and fitted with a HP-5MS column (30 m by 0.25 mm, df = 0.25 µm). The GC settings were as follows: initial oven temperature was held at 60°C for 1 min, and then ramped at 10°C/min to 180°C for 1 min and then ramped at 20°C/min to 280°C for 15 min. The injector temperature was maintained at 270°C. The samples (1 µl) were injected neat, with a split ratio of 1:10. The spectra were scanned from 20 to 550 *m/z* at two scans s^−^^1^. Most constituents were identified by GC by comparison of their retention indices with those of the literature (Rui and Ji 1992, Wang et al. 1994, Shi et al. 2011) or with those of authentic compounds available in our libraries (*Wiley* and *NIST* databases). The retention indices were determined in relation to a homologous series of *n*-alkanes (C_8_-C_24_) under the same operating. Component relative percentages were calculated based on GC peak areas for each component.

### Bioassay-Directed Fractionation

The crude essential oil (7 ml) was chromatographed on a silica gel column (3 cm in i.d., 35 cm in length) by gradient elution with a mixture of solvents (petroleum ether, petroleum ether-ethyl acetate, from 100:1,…, 100:50). Fractions of 250 ml were collected and concentrated at 40°C, and similar fractions were combined to yield 30 fractions according to thin layer chromatography profiles. Fractions 2–4 that possessed contact/repellency toxicity, with similar thin layer profiles, were polled and further purified by preparative silica gel column chromatography to obtain one pure compound for determining structure as p-cymene-8-ol (0.8 g). Fraction 13 that possessed contact and repellency toxicity was further purified by preparative silica gel column chromatography to obtain myristicin (1.7 g). ^1^H- and ^13^C-nuclear magnetic resonance (NMR) data were recorded on Bruker Avance DRX 500 NMR spectrometer with TMS (Tetramethylsilane) as the internal standard using deuterochloroform (CDCl_3_) as the solvent.

### Contact Toxicity

The contact toxicity of essential oil against *L. serricorne* adults was measured as described by Liu and Ho (1999)*.* Range-finding studies were run to determine the appropriate testing concentrations. A serial dilution of the essential oil was prepared in *n*-hexane. Aliquots of 0.5 μl of the dilutions were applied topically to the dorsal thorax of the insects. Both treated and control insects were then transferred to glass vials (10 insects per vial) with culture medium and kept in incubators. The mortality of the insects was observed after 24 h. The LD_50_ values were calculated by using probit analysis (Sakuma 1998). The positive control, pyrethrum extract (25% pyrethrine I and pyrethrine II), was purchased from Fluka Chemie, St.Louis, USA.

The contact toxicity of the essential oil against *Li. bostrychophila* was measured as described by Zhou et al. (2012). A 5.5 cm diameter filter paper was treated with 150 µl of the solution of the essential oil. The filter paper after being treated with solid glue was placed in a 5.5 cm diameter Petri dish and 10 booklice were put on the filter paper. A cover was put and all the Petri dishes were kept in incubators. *n*-Hexane was used as a negative control. Five concentrations (in *n*-hexane) and five batches of each concentration were used. The experiment was repeated twice. Mortality of insects was observed after 24 h. The LD_50_ values were calculated by using Probit analysis (IBM SPSS V20.0) (Sakuma 1998).

### Repellent Toxicity

A commercial repellent, DEET (*N, N*-diethyl-3-methyl benzamide) was purchased from National Center of Pesticide Standards (Shenyang, China) and used as a positive control. Petri dishes 6 cm in diameter were used to confine *Li. bostrychophila* during the experiment. The essential oil of *C. anisum-olens* and the isolated compounds were diluted in *n*-hexane to different concentration (31.58, 6.32, 1.26, 0.25, 0.05 nl/cm^2^), and *n*-hexane was used as the control. Filter papers, 6 cm in diameter, were cut in half and 150 μl of each concentration was applied separately to one-half of the filter paper as uniformly as possible with a micropipette. The other half (control) was treated with 150 μl of *n*-hexane. Both the treated and the control half were then air dried to evaporate the solvent completely (30 s). A full disc was carefully remade by attaching the tested half to the negative control half with tape. Each remade filter paper after being treated with solid glue was placed in a Petri dish. The solid glue was used to attach filter paper to the dish. Twenty insects were released in the center of each filter paper disc, and a cover was placed over the Petri dish. Five batches were used, and the experiment was repeated twice. Counts of the insects present on each strip were made after 2 h and 4 h. The percent repellency of each volatile oil or compound was then calculated using the formula:

$$\text{PR}\left(\%\right)=[(N_{c}-N_{t})/(N_{c}+N_{t})]\times \text{1}00,$$

where *N*_c_ was the number of insects present in the negative control half, while *N*_t_ was the number of insects present in the treated half.

The averages were then assigned to different classes (0–V) using the following scale (percentage repellency) (Liu and Ho 1999). Class, % repellency: 0, >0.01 to <0.1; I, 0.1–20.0; II, 20.1–40.0; III, 40.1–60.0; IV, 60.1–80.0; and V, 80.1–100.

In the experiments with *L. serricorne*, dishes and filter papers were changed to 9 cm in diameter and the concentration of the oils and isolated constituents were 39.32, 7.86, 1.57, 0.31, and 0.06 nl/cm^2^. The half filter paper was treated with 500 μl of the solution. Analysis of variance (ANOVA) and Tukey’s test were conducted by using SPSS 20.0 for Windows 2007. Percentage was subjected to an arcsine square-root transformation before ANOVA and Tukey’s tests.

## Results

### Chemical Composition of the Essential Oil

**Fig. 1.**
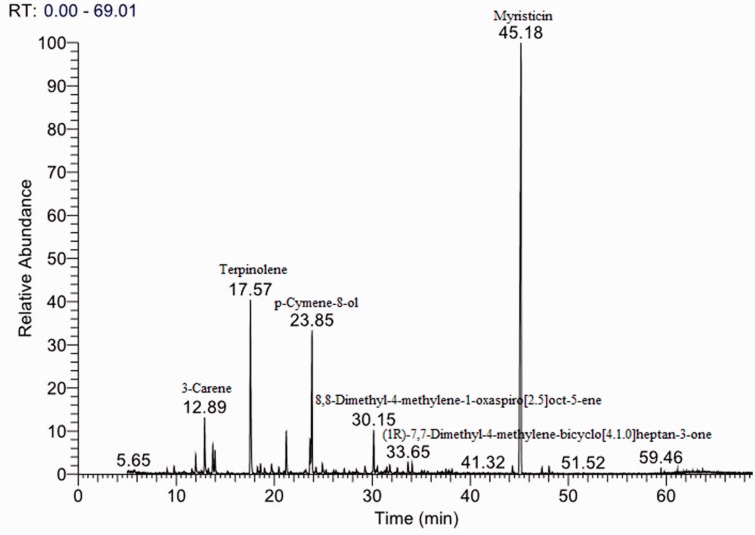
GC chromatograph of separated compounds from *C. anisum-olens* essential oil.

Yield of the *C. anisum-olens* essential oil from air-dried leaves was 0.35% (v/w). A total of 27 major components of the essential oil were identified, accounting for 84.29% of the total components present (Table 1). The main components were myristicin (36.87%), terpinolene (13.92%), and p-cymene-8-ol (12.38%) (Fig. 1).

**Table 1.** Chemical constituents of the essential oil derived from *C. anisum-olens* leaves

No.	Compounds	RI^*a*^	Chemical formula	Peak area (%)
1	α-Pinene	931	C_10_H_16_	0.27
2	α-Fenchene	938	C_10_H_16_	0.14
3	Myrcene	988	C_10_H_16_	1.16
4	α-Phellandrene	999	C_10_H_16_	0.19
5	3-Carene	1,004	C_10_H_16_	4.07
6	Isoterpinolene	1,063	C_10_H_16_	0.34
7	o-Cymene	1,070	C_10_H_14_	1.92
8	β-Terpinyl acetate	1,079	C_12_H_20_O_2_	1.56
9	Terpinolene	1,083	C_10_H_16_	13.92
10	Benzylin	1,138	C_15_H_14_O_3_	0.22
11	1,5,8-p-Menthatriene	1,142	C_10_H_14_	2.27
12	4-methylacetophenone	1,150	C_9_H_10_O	2.13
13	p-Cymene-8-ol	1,183	C_10_H_14_O	12.38
14	1-(4-Methylphenyl)ethanol	1,202	C_9_H_12_O	0.77
15	Cinerone	1,227	C_10_H_14_O	0.29
16	1-Cyclopentylcyclopentene	1,258	C_10_H_16_	0.17
17	8,8-Dimethyl-4-methylene-1-oxaspiro[2.5]oct-5-ene	1,286	C_10_H_14_O	1.65
18	2,6-Dimethyl-3,5,7-octatriene-2-ol	1,306	C_10_H_16_O	0.57
19	6-Isopropylidene-bicyclo[3.1.0]hexane	1,321	C_9_H_14_	0.28
20	4,4a,5,6-Tetrahydro-4, 7-dimethyl- cyclopenta[c]pyran-1,3-dione	1,355	C_10_H_13_O_3_	0.53
21	1-(1,1-Dimethylethoxy)-2-methyl-1-cyclohexene	1,372	C_11_H_20_O	0.45
22	(1R)-7,7-Dimethyl-4-methylene-bicyclo[4.1.0]heptan-3-one	1,396	C_10_H_14_O	0.16
23	Methyl eugenol	1,401	C_11_H_14_O_2_	0.34
24	Myristicin	1,489	C_11_H_12_O_3_	36.87
25	β-Bisabolene	1,504	C_15_H_24_	0.55
26	Elemicin	1,551	C_12_H_16_O_3_	0.50
27	Spathulenol	1,563	C_15_H_24_O	0.59
	Total			84.29

^*a*^RI, retention index as determined on a HP-5MS column using the homologous series of *n*-hydrocarbon.

### Isolated Compounds

**Fig. 2.**
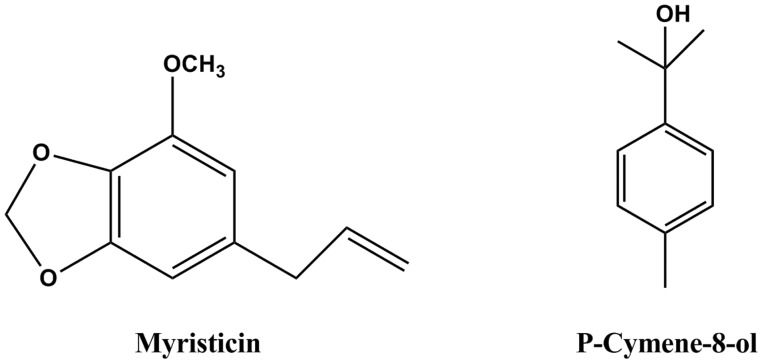
Structure of myristicin and p-cymene-8-ol isolated from *C. anisum-olens* essential oil.

**Fig. 3.**
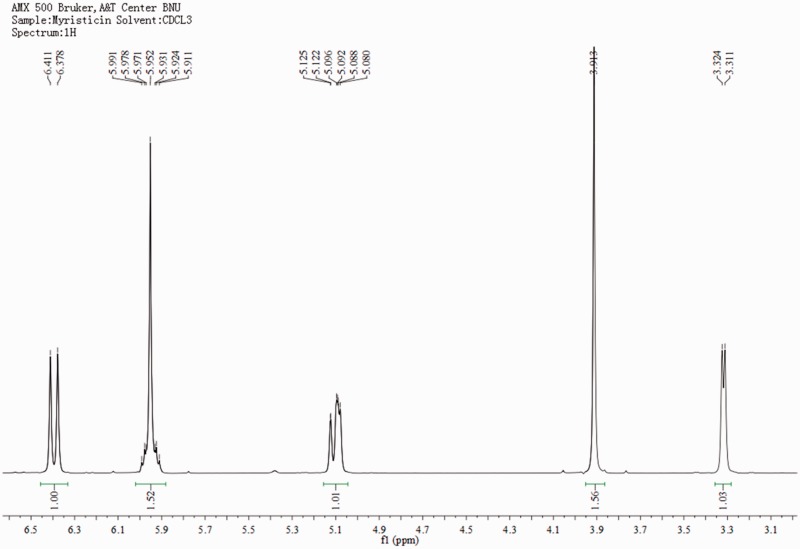
The 1H-NMR spectra of myristicin.

**Fig. 4.**
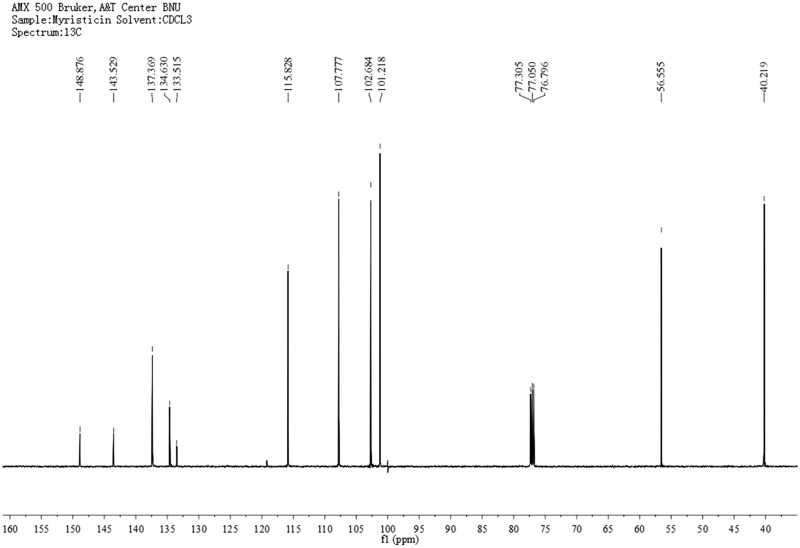
The 13C-NMR spectra of myristicin.

\Based on bioassay-guided fractionation, two compounds were separated and purified by column chromatography and preparative thin layer chromatography. The identifications were supported by the following data: Myristicin (1, Fig. 2), slightly yellow oil, C_11_H_12_O_3_. ^1^H-MNR (500 MHz, CDCl_3_) δ: 3.32 (2H, d, *J* = 6.5 Hz, 1-CH_2_), 3.91 (3H, s, 3'-OCH_3_), 5.09 (2H, m, 3-CH_2_), 5.95 (2H, s, O-CH_2_-O), 5.96 (1H, m, 2-H), 6.38 (1H, s, 6'-H), 6.41(1H, s, 2'-H). ^13^C-NMR (125 MHz, CDCl_3_) δ: 40.2 (C-1), 56.6 (C-OCH_3_), 101.2 (O-CH_2_-O), 102.7 (C-6'), 107.8 (C-2'), 115.8 (C-3), 133.5 (C-1'), 134.6 (C-4') 137.3 (C-2), 143.5 (C-3'), 148.8 (C-5'). ^1^H- and ^13^C-NMR spectra are presented in Figures 3 and 4. Its NMR data were in accord with the reported data of Passreiter et al. (2005).

**Fig. 5.**
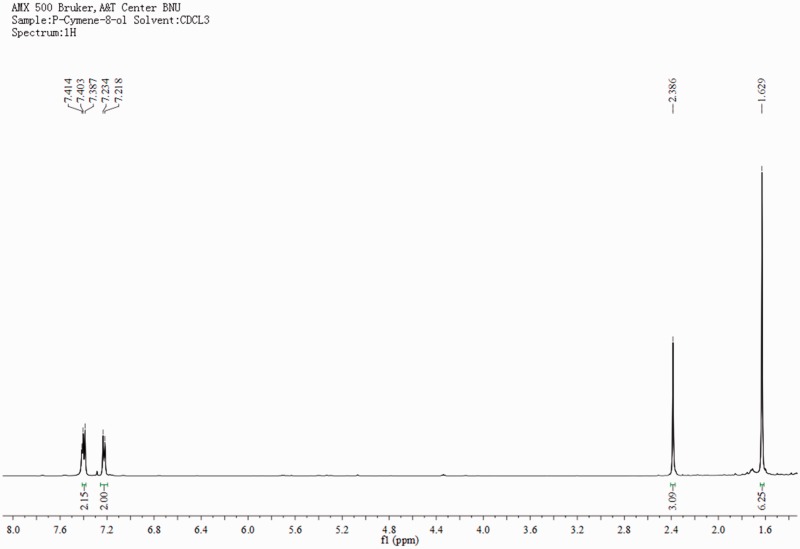
The 1H-NMR spectra of p-cymene-8-ol.

**Fig. 6.**
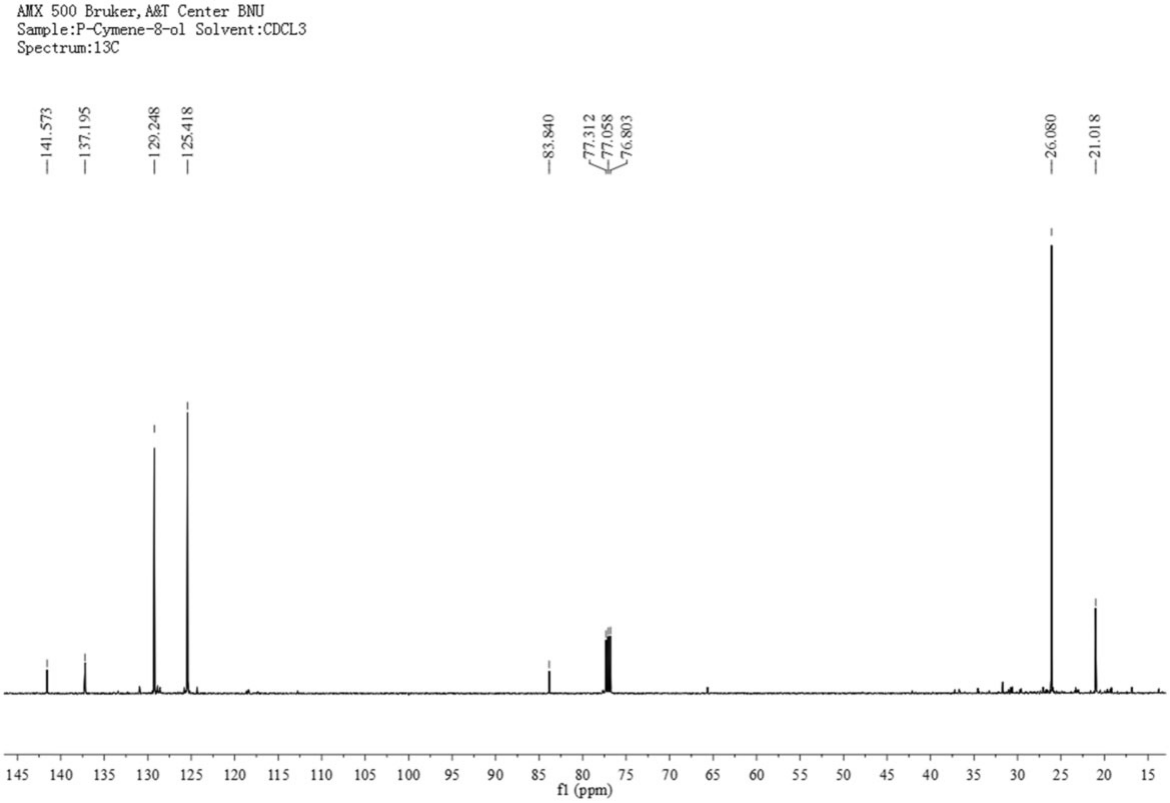
The 13C-NMR spectra of p-cymene-8-ol.

P-Cymene-8-ol (2, Fig. 2), slightly yellow oil, C_10_H_14_O. ^1^H-MNR (500 MHz, CDCl_3_) δ: 1.63 (6H, s, CH_3_-C-CH_3_), 2.39 (3H, s, 1-CH_3_), 7.23 (2H, d, *J* = 8.0 Hz, 3-H and 5-H), 7.41 (2H, d, *J* = 8.0 Hz, 2-H and 6-H). ^13^C-NMR (125 MHz, CDCl_3_) δ: 21.0 (C-CH_3_), 26.1 (CH_3_-C-CH_3_), 83.8 (C-1'), 125.4 (C-3 and C-5), 129.2 (C-2 and C-6), 137.2 (C-1), 141.6 (C-4). ^1^H- and ^13^C-NMR spectra are presented in Figures 5 and 6. The above data were identical to the published data of Gurudutt et al. (1984) and Yamamoto et al. (2011).

### Insecticidal Toxicity

Myristicin showed stronger contact toxicity against *L. serricorne* (LD_50_ = 18.96 μg per adult) and *Li. bostrychophila* (LD_50_ = 20.41μg per adult) than p-cymene-8-ol (LD_50_ = 39.68 and 35.66 μg per adult, respectively), while the essential oil of *C. anisum-olens* possessed contact toxicity against *L. serricorne* and *Li. bostrychophila* with LD_50_ values of 12.44 μg per adult and 74.46 μg per adult, respectively. However, when compared with the positive control, the essential oil of *C. anisum-olens* had 52 and 4 times less acute toxicity against the two species of grain storage insects (Tables 2 and 3).

**Table 2.** Contact toxicity of the essential oil of *C. anisum-olens* against *L. serricorne*

Insects	Treatments	LD_50_ (μg/adult)	95% fiducial limits	χ^2^
*L. serricorne*	*C. anisum-olens*	12.44	6.37 ± 17.14	17.36
	Myristicin	18.96	16.20 ± 21.75	19.78
	p-cymene-8-ol	39.68	33.47 ± 45.55	16.79
	Pyrethrum extract	0.24	0.16 ± 0.35	16.33

**Table 3.** Contact toxicity of the essential oil of *C. anisum-olens* against *Li. bostrychophila*

Insects	Treatments	LD_50_ (μg/cm^2^)	95% fiducial limits	χ^2^
*Li. bostrychophila*	*C. anisum-olens*	74.46	67.16–79.31	21.16
	myristicin	20.41	18.81–22.07	20.93
	p-cymene-8-ol	35.66	32.62–39.07	19.32
	Pyrethrum extract	18.99^*a*^	—	—

^*a*^Data from Liu et al. (2013b).

### Repellent Activity

The results of repellency assays for the essential oil and isolated constituents against the two species of insects were presented in Tables 4 and 5. Data showed that *C. anisum-olens* exhibited strong repellency against *L. serricorne* and *L.* *bostrychophila*. At the test concentration of 39.32 nl/cm^2^, the essential oil and the positive control, DEET showed the same level repellency against *L. serricorne* at 2 h/4 h after exposure, whereas at the test concentrations of 1.57 and 0.31 nl/cm^2^, the essential oil exhibited stronger repellency (38% and 32%, respectively) than DEET (28% and 20%, respectively) against *L. serricorne* at 2 h after exposure*.* However, at the test concentrations of 39.32 and 6.32 nl/cm^2^, the essential oil and DEET also showed the same level repellency against *Li. bostrychophila* at 2 h/4 h after exposure (class V).

**Table 4.** Percentage repellency after two exposure times for the essential oil and isolated constituents against *L. serricorne*^*a*^ (df = 3)

Treatment	2 h					4 h				
	39.32^*b*^	7.86^*b*^	1.57^*b*^	0.31^*b*^	0.06^*b*^	39.32^*b*^	7.86^*b*^	1.57^*b*^	0.31^*b*^	0.06^*b*^
*C. anisum-olens*	96 ± 4a	54 ± 12bc	38 ± 10b	32 ± 7a	12 ± 7a	86 ± 6b	30 ± 9c	10 ± 6b	2 ± 3b	−26 ± 6c
Myristicin	78 ± 5bc	72 ± 9ab	68 ± 10a	4 ± 3b	−18 ± 7b	80 ± 12b	50 ± 6b	−8 ± 5c	−46 ± 7c	−60 ± 6d
p-cymene-8-ol	64 ± 9c	46 ± 4c	22 ± 3b	14 ± 4ab	6 ± 4a	58 ± 9c	50 ± 10b	16 ± 9b	8 ± 7b	4 ± 3b
DEET	88 ± 7ab	76 ± 14a	28 ± 7b	20 ± 14ab	16 ± 7a	98 ± 4a	78 ± 9a	58 ± 16a	56 ± 14a	46 ± 7a
*F*	10.347	4.412	12.318	3.575	26.006	8.759	13.803	21.096	78.738	165.515
*P*	0	0.019	0	0.038	0	0.001	0	0	0	0

^*a*^Means in the same column followed by the same letters do not differ significantly (*P* < 0.05) in ANOVA. PR was subjected to an arcsine square-root transformation before ANOVA.

^*b*^Concentration (nl/cm^2^).

**Table 5.** Percentage repellency after two exposure times for the essential oil and isolated constituents against *Li. bostrychophila*^*a*^ (df = 3)

Treatment	2 h					4 h				
	31.58^*b*^	6.32^*b*^	1.26^*b*^	0.25^*b*^	0.05^*b*^	31.58^*b*^	6.32^*b*^	1.26^*b*^	0.25^*b*^	0.05^*b*^
*C. anisum-olens*	94 ± 3b	86 ± 4b	42 ± 8c	22 ± 5b	14 ± 7a	92 ± 8a	68 ± 7b	32 ± 8c	16 ± 15b	12 ± 10ab
Myristicin	90 ± 4b	100 ± 0a	94 ± 3a	54 ± 7a	22 ± 10a	96 ± 3a	98 ± 3a	92 ± 7a	20 ± 18b	6 ± 10b
p-cymene-8-ol	100 ± 0a	96 ± 7a	32 ± 11c	22 ± 11b	18 ± 7a	96 ± 7a	74 ± 16b	30 ± 10c	18 ± 11b	16 ± 4ab
DEET	100 ± 0a	98 ± 3a	78 ± 10b	66 ± 8a	8 ± 3a	96 ± 3a	82 ± 5b	68 ± 3b	54 ± 6a	22 ± 5 a
*F*	6.806	8.058	24.249	15.694	1.491	0.149	7.592	27.069	2.979	2.383
*P*	0.004	0.002	0	0	0.255	0.929	0.002	0	0.063	0.108

^*a*^Means in the same column followed by the same letters do not differ significantly (*P* < 0.05) in ANOVA. PR was subjected to an arcsine square-root transformation before ANOVA.

^*b*^Concentration (nl/cm^2^).

Myristicin and p-cymene-8-ol had strong repellent toxicity against *Li.* *bostrychophila* and moderate repellency against *L. serricorne*. Compared with the positive control, at the concentration of 1.26 nl/cm^2^, myristicin exhibited stronger repellency (92%, class V) than DEET (68%, class IV) against *Li. bostrychophila* at 4 h after exposure. Moreover, at the concentration of 1.57 nl/cm^2^, myristicin showed strongly repellent (68%, class IV) against *L. serricorne,* while the positive control showed less repellency (28%, class II) at 2 h after exposure. When at the test c, p-cymene-8-ol possessed the same level repellency against *Li. bostrychophila* relative to DEET at 2 h and 4 h after exposure (class V).

## Discussion

### Chemical Composition of the Essential Oil

The main components of the essential oil of *C. anisum-olens* were myristicin (36.87%), terpinolene (13.92%), and p-cymene-8-ol (12.38%). The chemical composition of the essential oil of *C. anisum-olens* in this study was not the same as what had been reported in previous studies. For example, Su and Liang (2011) found that the essential oil of *C. anisum-olens* leaves collected from Fusui of Guangxi Province contained myristicin (28.97%) and (+) - 4 - carene (24.84%). Moreover, there were some variations in the essential oil derived from different parts of *C. anisum-olens*. Su et al. (2011) indicated that the major components of the essential oil of *C. anisum-olens* nutlets harvested from Longzhou of Guangxi Province were myristicin (47.07%), 1,2,3-trimethoxy-5-(2-propenyl)-benzene (8.25%), 2,6-dimethoxy-4-(2-propenyl)-pheno (7.17%), and *n*-hexadecanoic acid (7.05%). These differences of chemical content and composition of the essential oils might have been due to different parts, harvest time, local, season factors, and storage duration, and these differences may result in different biological activities. The above results suggested further studies on plant cultivation and essential oil standardization were needed.

### Insecticidal Toxicity

The essential oil of *C. anisum-olens* showed stronger contact toxicity against *L. serricorne* than the isolated compounds, which had almost 1.5 and 3 times more toxicity than myristicin and p-cymene-8-ol against *L. serricorne*. The results indicated that the contact toxicity of the essential oil against *L. serricorne* was related to the synergistic effects of its diverse major and minor components. Myristicin had almost 2 and 1.7 times more toxicity than p-cymene-8-ol against *L. serricorne* and *Li. bostrychophila*, respectively. It is suggested that myristicin is a major contributor to the contact toxicity of the essential oil against *L. serricorne* and *Li. bostrychophila*.

The essential oil showed weaker contact toxicity against *L. serricorne* and *Li. bostrychophila* than the positive control. However, when compared with other essential oils, *C. anisum-olens* essential oil possessed stronger contact toxicity against the two stored product insects, e.g., essential oils of *Litsea cubeba* (LD_50_ = 27.33 μg per adult) against *L. serricorne* (Yang et al. 2014), *Cinnamomum camphora* (LD_50_ = 21.25 μg per adult) against *L. serricorne* (Chen et al. 2014), *Acorus calamus* (LD_50_ = 100.21 μg per cm^2^) against *Li. bostrychophila* (Liu et al. 2013b), and *Artemisia rupestris* (LD_50_ = 418.48 μg per adult) against *Li. bostrychophila* (Liu et al. 2013a).

As currently used insecticides are generally synthetic and the most effective of which (e.g., phosphine and methyl bromide) are also highly toxic to humans and other nontarget organisms. The essential oil of *C. anisum-olens* leaves and its active compounds show potential to be developed into possible natural insecticides for the control of two stored product insects. However, the dose rates of the essential oil and isolated compounds used here were high when compared with the concentrations of the positive control. So the practical application of the essential oil and the isolated compounds as novel insecticides, further studies on the safety of the essential oil to humans and on development of formulations are necessary to improve the efficacy and stability and to reduce costs.

### Repellent Activity

The essential oil and isolated compounds showed stronger repellent activity against *Li. bostrychophila* than *L. serricorne*. At the concentration of 31.58 nl/cm^2^, the percentage repellency against *Li. bostrychophila* of the essential oil and isolated compounds were even higher than 90% at 2 and 4 h after exposed. So *Li. bostrychophila* was more sensitive to the essential oil and isolated compounds than *L. serricorne*. Moreover, it was found that the variety of the percentage repellency was obviously affected by the testing concentrations and the exposure duration. The percentage repellency of the essential oil and the two compounds increased with the increase of testing concentrations except that myristicin showed strongest repellent activity against *Li. bostrychophila* at the concentration of 6.32 nl/cm^2^. So the recommended concentration of the essential oil and p-cymene-8-ol against *L. serricorne* and *Li. bostrychophila* is 39.32 nl/cm^2^, whereas 39.32 nl/cm^2^ is the recommended concentration of myristicin against *L. serricorne* and 6.32 nl/cm^2^ is the recommended concentration of myristicin against *Li. bostrychophila*.

In this article, we reported the isolation of two insecticidal constituents from the essential oil of *C. anisum-olens* for the first time. The work indicates that the *C. anisum-olens* essential oil and its isolated constituents possess significant contact and repellent activities against two stored product insects. As rich in natural resources, *C. anisum-olens* essential oil and bioactive compounds have potential to apply in controlling stored product pests. However, for the practical application of the essential oil and the isolated constituents in real-world pest control, further studies on the safety, efficacy, and stability are necessary.
